# Essential health information available for India in the public domain on the internet

**DOI:** 10.1186/1471-2458-9-208

**Published:** 2009-06-29

**Authors:** Magdalena Z Raban, Rakhi Dandona, Lalit Dandona

**Affiliations:** 1 School of Public Health and George Institute for International Health, University of Sydney, Sydney, Australia; 2 George Institute for International Health, India, Hyderabad, India; 3 Institute for Health Metrics and Evaluation, University of Washington, Seattle, Washington, USA

## Abstract

**Background:**

Health information and statistics are important for planning, monitoring and improvement of the health of populations. However, the availability of health information in developing countries is often inadequate. This paper reviews the essential health information available readily in the public domain on the internet for India in order to broadly assess its adequacy and inform further development.

**Methods:**

The essential sources of health-related information for India were reviewed. An extensive search of relevant websites and the PubMed literature database was conducted to identify the sources. For each essential source the periodicity of the data collection, the information it generates, the geographical level at which information is reported, and its availability in the public domain on the internet were assessed.

**Results:**

The available information related to non-communicable diseases and injuries was poor. This is a significant gap as India is undergoing an epidemiological transition with these diseases/conditions accounting for a major proportion of disease burden. Information on infrastructure and human resources was primarily available for the public health sector, with almost none for the private sector which provides a large proportion of the health services in India. Majority of the information was available at the state level with almost negligible at the district level, which is a limitation for the practical implementation of health programmes at the district level under the proposed decentralisation of health services in India.

**Conclusion:**

This broad review of the essential health information readily available in the public domain on the internet for India highlights that the significant gaps related to non-communicable diseases and injuries, private health sector and district level information need to be addressed to further develop an effective health information system in India.

## Background

Health information and statistics are important for planning, monitoring and improvement of services for the health of populations [1]. These are essential for policymakers and programme planners to inform their decisions about what actions to take and what services to provide in order to improve the health of the populations they serve. Though developing countries account for the majority of the global burden of disease, the availability of health information is not adequate in many of these countries [1-3]. The lack of quality health information has become more apparent in recent years with the Millennium Development Goals and demand from international organisations for monitoring and evaluation data on health programmes supported by them [4].

The Government of India in its National Health Policy of 2002 acknowledged the absence of systematic and scientific population health statistics as a major deficiency in India [5]. There have been a few recent efforts to strengthen some of the data sources in India [6-9]. While the data sources themselves require strengthening, it is important that the information generated by them is made available in the public domain in order for it to be utilised by a variety of stakeholders. In this background, as an initial step we reviewed the essential health information readily available in the public domain on the internet for India.

## Methods

We reviewed the essential sources of health-related information for India as identified by AbouZahr and Boerma [10]. The search strategy included extensive searches of the various websites of the Government of India and the relevant national and international organisations using Google [11] and search of the PubMed literature database [12].

### Identification of available data

The ten essential sources of information included in this report are: census; birth and death registration; surveillance and response systems; household surveys; service generated data; mapping of health facilities; behavioural surveillance; national health accounts; financial and management information; and modelling, estimates and projections [10]. These information sources provide data at a variety of levels household, patient, health facility, district, state and national. Health research, one of the other essential sources of information [10], was not included in this review as it is a major topic by itself and we have previously reported on health research output from India [13].

As a first step, extensive searches were carried out for the essential sources and their outputs on websites of the government, national health programmes, nongovernmental institutions, and international agencies relevant for the health information system in India. These searches provided further useful links to other relevant websites that provided information on essential sources of population health information in India. In addition, for each essential source a Google search was carried out using "India" AND the essential source related terms. For example, for birth and death registration, the search combinations of "birth registration, death registration, or sample registration system AND India" were used. A similar strategy was used to search PubMed for publications 1990 onwards on essential sources of health information in India. From these various searches, the organisations or programmes that yielded usable information regarding the content, process or outputs of essential sources of health information for India are shown in Table 1.

**Table 1:** Sources that yielded usable essential health information for India

**Organisation**	**Website**
Census of India	
Central Bureau of Health Intelligence	
Centre for Global Health Research	
Department of Family Welfare	
Disease Control Priorities Project	
Jansankhya Sthirata Kosh (National Population Stabilisation Fund)	
Ministry of Health and Family Welfare	
Ministry of Statistics and Programme Implementation	
National AIDS Control Organisation	
National Cancer Registry Programme	
National Commission on Population	
National Crime Records Bureau	
National Family Health Survey, India	
National Institute of Nutrition	
National Polio Surveillance Project	
National Programme for the Control of Blindness	
National Sample Survey Organisation	
National Vector Borne Disease Control Programme	
Planning Commission	
Reproductive and Child Health District Level Household and Facility Surveys	
Tuberculosis Control in India	
United Nations Children's Fund – Child info	
World Bank – Living Standards Measurement Study	
World Health Organisation World Health Survey	
World Health Organization India Country Office	
World Health Organization South East Asia Regional Office	

### Assessment of available data

For each essential information source, the periodicity of data collection, the information it generated, the geographical level at which this information is reported, and the extent of its availability in the public domain were assessed. Additional searches and reviews were conducted, if needed, of the relevant reports and publications accessible in the public domain to assist with these assessments.

*Periodicity *was defined as the frequency with which data collection was carried out. The latest year of data collection is reported. The *information generated *by each essential source was classified into these five categories: mortality and causes of death, morbidity and health status, risk factors, service provision, and health resources [4]. Information on health resources was sub-categorised based on whether it was related to infrastructure, human resources or financing [14].

The available information was further assessed to understand how it related to the major causes of disease burden in India as estimated by the Global Burden of Disease Project [15]. The rationale for the use of the leading causes of disease burden was based on the idea that relevant health information for these causes at least should be available. All available information produced by the essential sources was examined. Mortality, morbidity, risk factor and service provision information related to the conditions was listed. A risk factor for each condition was considered if it was listed in the major publications examining risk factors or a review of risk factors in India [16-18]. Service provision indicators considered were the interventions that addressed the risk factors or treatment of a disease or condition.

*Geographical level of information reported *was assessed for the administrative level for which the information was available: national, state, district or city/town. The extent of *availability in the public domain *on the internet was rated based on whether all the information produced was freely available including reports, microdata and metadata where appropriate (+++); all types of information was available but with restrictions such as purchase cost (++); only some information was available, for example reports but no microdata (+); or no information was available in the public domain (0). Availability of microdata was not used as a criterion for the documents or sources that dealt with secondary data or were based on other sources.

## Results

The findings related to the characteristics of health information for India available from essential sources in the public domain on the internet are summarized in Table 2[6,8,9,16,19-57].

**Table 2:** Characteristics of essential sources of health information in India, 2008.

**Essential source **[10]	**Year started**	**Periodicity; last year conducted**	**Category of information**	**Lowest geographical level**	Availability of information in the public domain*
**1. Census **[19]	1872	10 years; 2001	◦ Morbidity & health status	City/town/village	+
			◦ Risk factors		
			◦ Health infrastructure		

**2. Birth and death registration**					

a) National birth and death registration system [20-22]	1969	Ongoing	◦ Mortality & causes of death	State	+
			◦ Morbidity & health status		

b) Sample registration system [6,23]	1969	Ongoing	◦ Mortality & causes of death	State	+
			◦ Morbidity & health status		

**3. Surveillance and response system**					

a) HIV Sentinel Surveillance [8,24]	1998	Annual; 2007	◦ Morbidity & health status	District†	+

b) National Crime Records Bureau "Accidental deaths and suicides in India" [25]	1967	Annual; 2007	◦ Mortality & causes of death	State	+

c) National Cancer Registry Programme [26]	1981	Variable; 2003	◦ Mortality & causes of death	City‡	+
			◦ Morbidity & health status		
			◦ Service provision		

d) Integrated Disease Surveillance Project [9]	2003	Ongoing	◦ Mortality & causes of death	Not known	0
			◦ Morbidity & health status		

e) National Polio Surveillance Project [27]	1997	Ongoing	◦ Morbidity & health status	State	+++

f) National Vector Borne Diseases Control Programme [28]	Not available	Ongoing	◦ Mortality & causes of death	State	+
			◦ Morbidity & health status		

**4. Household surveys**					

a) Reproductive and Child Health District Level Household Surveys [29]	1998	4–5 years; 2007/8	◦ Morbidity & health status	District§	+++§
			◦ Risk factors		
			◦ Service provision		

b) National Family Health Surveys [30]	1990	6–7 years; 2005/6	◦ Mortality	State	+++
			◦ Morbidity & health status		
			◦ Risk factors		
			◦ Service provision		

c) National Sample Survey on household consumer expenditure [31]	1972	5 years for large samples (surveys on small samples since 1986/7); 2005/6	◦ Health financing	State	++

d) National Sample Survey on nutritional intake in India [32]	1972	5–6 years; 2004/5	◦ Morbidity & health status	State	++
			◦ Risk factors		

e) National Sample Survey on morbidity, health care and the condition of the aged [33]	1973	Variable (conducted twice); 2004	◦ Morbidity & health status	State	++
			◦ Risk factors		
			◦ Service provision		

f) World Health Survey [34]	2003	Once; 2003	◦ Mortality & causes of death	State||	+++
			◦ Morbidity & health status		
			◦ Risk factors		
			◦ Service provision		
			◦ Human resources		

g) National Sample Survey on condition of urban slums [35]	1976	Variable (conducted thrice); 2002	◦ Risk factors	State	++
			◦ Health infrastructure		

h) National Sample Survey on disabled persons in India [36]	1981	10–11 years; 2002	◦ Morbidity & health status	State	++

i) National Sample Survey report on village facilities [37]	1950	Variable; 2002	◦ Risk factors	State	++
			◦ Health infrastructure		

j) UNICEF Multiple Indicator Survey [38]	2000	Once; 2000	◦ Morbidity & health status	State	+
			◦ Risk factors		
			◦ Service provision		

k) National Sample Survey on drinking water, sanitation and hygiene [39]	1988	Variable (conducted thrice); 1998	◦ Risk factors	State	++

l) World Bank Survey of Living Conditions in Uttar Pradesh and Bihar [40]	1997/8	Once; 1997/8	◦ Morbidity & health status	State¶	+
			◦ Risk factors		
			◦ Service provision		

m) National Sample Survey on maternal and child health care in India [41]	1995/6	Once; 1995/6**	◦ Risk factors	State	++
			◦ Service provision		

n) National Sample Survey on consumption of tobacco in India, 1993–94 [42]	1993/4	Once; 1993/4**	◦ Risk factors	State	++

**5. Service generated data**					

a) Family Welfare Statistics in India [43]	1974	Variable; 2006	◦ Service provision	State	+

b) Performance of Cataract Surgery under National Programme for Control of Blindness [44]	Not available	Ongoing	◦ Service provision	State	+

c) Revised National Tuberculosis Control Programme [45]	1997	Ongoing	◦ Service provision	District††	+++
			◦ Health infrastructure		
			◦ Human resources		

**6. Mapping of health facilities**					

a) Bulletin on Rural Health Statistics [46]	Not available	Annual; 2007	◦ Health infrastructure	State; some district	+
			◦ Human resources		

b) National Health Profile [47]	1953	Annual; 2007	◦ Morbidity & health status	State	+++¶¶
			◦ Health infrastructure		
			◦ Human resources		

c) WHO "Antiretroviral Therapy Service Map" [48]	2006	Once; 2006	◦ Service provision	State	+

d) Reproductive and Child Health Facility Survey [49]	1998	5 years; 2003	◦ Service provision	State	+++‡‡
			◦ Health infrastructure		
			◦ Human resources		

e) Jansankhya Sthirata Kosh & National Informatics Centre health maps [50]	2007§§	Once; 2001	◦ Health infrastructure	District	+++¶¶

**7. Behavioural surveillance**					

a) NACO Behavioural Surveillance Surveys [51]	2001	5 years; 2006	◦ Risk factors	State	+
			◦ Service provision		

b) National Nutrition Monitoring Bureau time trend surveys [52]	1972	8–13 years; 1996/7	◦ Risk factors	State||||	+

c) Integrated Disease Surveillance Project [9]	2003	Ongoing	◦ Risk factors	Not known	0

d) Sample Registration System [23,53]	2001	Ongoing	◦ Risk factors	State	+***

**8. National Health Accounts **[54]	2001/2	3 years; 2004/5†††	◦ Health financing	State	+++

**9. Financial and management information**					

a) Ministry of Health and Family Welfare "Annual Report" [55]	Not available	Annual; 2007/8	◦ Health infrastructure	National	+++¶¶
			◦ Human resources		
			◦ Health financing		

b) Planning Commission "Five Year Plan" [56]	1950	5 years; 2007	◦ Health infrastructure	National	+++¶¶
			◦ Human resources		
			◦ Health financing		

c) National Commission on Macroeconomics and Health [57]	2005	Once; 2005	◦ Health infrastructure	State	+++¶¶
			◦ Human resources		
			◦ Health financing		

**10. Modelling, estimates and projections**					

a) National Commission on Macroeconomics and Health [57]	2004	Once; 2005	◦ Mortality & causes of death	National;	+++¶¶
			◦ Morbidity & health status	some state	
			◦ Health infrastructure		
			◦ Human resources		
			◦ Health financing		

b) Global Burden of Disease and Risk Factors Study [16]	1990	Variable; 2002‡‡‡	◦ Mortality & causes of death	National	+++
			◦ Morbidity & health status		
			◦ Risk factors		

*Availability of information: +++ means all types of information produced freely available (reports, details, microdata and metadata as applicable), ++ all types available but with restrictions, + means some available (e.g. report or spreadsheet, but no microdata), and 0 means none available.

†At time of writing this paper, district level estimates available for 2006 for HIV prevalence among antenatal clinic attendees and patients with sexually transmitted diseases; estimates of state level population HIV prevalence available for 2007.

‡Only for registry locations; population-based registries at Ahmedabad, Aurangabad, Bangalore, Barshi, Bhopal, Chennai, Delhi, Dibrugarh, Guwahati, Imphal, Kollam, Kolkata, Mizoram, Mumbai, Nagpur, Pune, Sikkim, Silchar, Thiruvananthapuram; hospital-based registries at Bangalore, Chennia, Mumbai, Dibrugarh.

§At the time of writing this paper, reports, metadata and microdata available for district level for 2002–2004 survey; state level reports available for 2007/8 survey.

||Only for the states of Assam, Karnataka, Maharashtra, Rajasthan, Uttar Pradesh, West Bengal; national pooled estimates also available.

¶Only for the states of Uttar Pradesh and Bihar.

**Part of a regular household consumption survey, but these are the only reports on maternal and child health, and tobacco consumption available.

†† District level only for service provision; infrastructure and human resources information available for state level.

‡‡At the time of writing this paper, reports, metadata and microdata available for the 2003 survey.

§§Maps produced in 2007 using 2001 census data.

|||| Only for the states of Andhra Pradesh, Gujarat, Karnataka, Kerala, Madhya Pradesh, Maharashtra, Orissa, Tamil Nadu, Uttar Pradesh, West Bengal.

¶¶Only reports available; these are not primary data collection sources, so microdata not expected.

***Some results have been released in the form of a journal publication [53].

†††National Health Accounts for 2004/5 are being computed but the results not available at the time of writing this paper.

‡‡‡Regional estimates for 2004 were made available in 2008.

### Periodicity

The periodicity of data collection ranged from on-going (e.g. sample registration system [6]), annual (e.g. HIV sentinel surveillance [8]) to decennial (census [19]) (Table 2). Some data collection under the household surveys [41,42], mapping of health facilities [48,50] and financial and management information was carried out only once, including a number of international survey programmes [34,38,40] and reports such as the National Commission on Macroeconomics and Health [57].

### Information generated

The essential sources generate all five categories of information, i.e. *mortality and causes of death, morbidity and health status, risk factors, service provision*, and *health resources*, but to varying degrees (Table 2). *Mortality and cause of death *information is generated by birth and death registration [6,20], four surveillance systems [9,25,26,28], two household surveys [30,34] and modelling [16,57]. Information on *morbidity and health status *is generated by the census [19], monitoring of birth and deaths [6,20], surveillance and response systems [8,9,26-28], eight household surveys [29,30,32-34,36,38,40], mapping of health facilities [47] and both modelling, estimates and projections sources [16,57]. *Risk factor information *is generated by census [19], most household surveys [29,30,32-35,37-42], the behavioural surveillance systems [9,51-53] and modelling, estimates and projections [16]. *Service provision *information, such as coverage of interventions, is generated by a surveillance and monitoring system [26], household surveys [29,30,33,34,38,40,41], service generated data [43-45], two mapping of health facilities [48,49] and one behavioural surveillance system [51].

Information on health system infrastructure is generated by census [19], sources under household surveys [35,37], mapping of health facilities [46,47,49,50] and financial and management information [55-57] (Table 2). Much of this deals with the numbers of public health facilities and the equipment availability in these facilities. Estimates of the number of private sector hospitals and beds have been made in the Planning Commission's Five Year Plan [56].

The National Health Profile [47] and the Report of the National Commission on Macroeconomics and Health [57] provide information on the total number of human resources for health (physicians, nursing staff and other paramedical personnel). The WHO World Health Survey provides the estimates of total numbers of health workers, as assessed by asking respondents if they were employed in the health sector [34]. The Bulletin on Rural Health Statistics [46] and the Reproductive and Child Health Facility Survey [49] provide data on the numbers of health workers in public health facilities, including physicians, laboratory workers, nurses and midwives. Revised National Tuberculosis Control Programme provides programme specific data on human resources [45]. Information on medical education facilities, such as numbers of medical colleges, is available in the National Health Profile [47], Bulletin on Rural Health Statistics [46], Annual Report of the Ministry of Health and Family Welfare [55], Planning Commission [56], and Report of the National Commission on Macroeconomics and Health [57].

The most comprehensive source of financing information was the National Health Accounts (NHA), completed for the year 2001–2 and is currently underway for 2004–5 [54]. It provides data on the government and private expenditure on health [54]. The other sources of financing information include the National Sample Survey on household consumer expenditure [31], Annual Report of the Ministry of Health and Family Welfare [55], National Health Profile [47], Planning Commission [56] and Report of the National Commission on Macroeconomics and Health [57].

Table 3 shows the available information generated by the essential sources for the leading causes of disease burden in India, other than that generated by the modelling and projections [8,19,23,25,32-34,36,38,40-45,48,51,53,58-67]. The conditions listed were each estimated to have contributed more than 2% of the total disease burden in India in the year 2002 [15]. Importantly, the Global Burden of Disease and Risk Factors study provides mortality and morbidity estimates for India for all conditions, some of which are based on modelling and projections as primary data are not available for some conditions. It also provides estimates of the distribution of selected risk factors in the population, again some of which are modelled projections [15].

**Table 3:** Population health information available in the public domain on the leading causes of disease burden in India.*

**Disease or health condition** (% of total DALYs lost in 2002 in India [15])	**Category**	**Information available**	**Lowest geographical level available**	**Comments**
**1. Perinatal conditions** (9.7%)	**Mortality**	Perinatal mortality rate [23,58-60]†	State	Based on data from sample registration system and large scale household survey ‡ No cause of perinatal death data.
	
	**Morbidity & health status**	Prevalence of low birth weight [38,58,59,61]	District	Based on data from large scale household survey‡
	
	**Risk factors**	Anaemia among women [58,59,62]	District	Most estimates based on data from large scale household survey‡
			
		Underweight among women [34,58,59]	State	
			
		Contraception non-use [38,43,58-61,63]	District	
			
		Birth spacing [58-60]	State	
			
		Number of children ever born 5 or more [58-61,63]	District	
			
		Antenatal care received [33,34,38,41,43,58-61,63]	District	
			
		Skilled birth attendance [33,34,38,41,58-61,63]	District	
			
		Low birth weight [38,58,59,61]	District	
			
		Prevalence of postpartum complications [58-61,63]	District	
	
	**Service provision**	Contraception coverage [38,43,58-61,63]	District	Most estimates based on data from large scale household survey‡
			
		Antenatal care coverage [33,34,38,41,43,58-61,63]	District	
			
		Women receiving iron supplementation [38,41,43,58-61,63]	District	
			
		Skilled birth attendance coverage [33,34,38,41,58-61,63]	District	
			
		Postnatal care coverage [33,38,41,58,59]	State	

**2. Lower respiratory tract infections** (8.5%)	**Mortality**	None*		
	
	**Morbidity & health status**	Prevalence of cough in children [34,38,58-61,63]	State	Based on data from large scale household surveys‡
	
	**Risk factors**	Type of cooking fuel used [19,34,38,40,58-60,63]	District	Most estimates based on large scale household surveys‡
			
		Tobacco smoking [34,42,53,58]	State	
			
		Childhood underweight [58-60,62]	State	
			
		Breastfeeding practices [38,58-61,63]	District	
	
	**Service provision**	Treatment of childhood cough [34,38,58-61,63]	State	Based on data from large scale household surveys‡

**3. Ischaemic heart disease** (5.1%)	**Mortality**	Percent of adult deaths attributed to chest pain [34]	National	Estimate based on a small sample of verbal autopsy on sibling deaths§
	
	**Morbidity & health status**	Self-reported diagnosis with ischaemic heart disease [34]	State||	Estimate based on household survey conducted in 6 states||
	
		Self-reported hospitalisations for heart disease [33]	National	
	
	**Risk factors**	Prevalence of overweight & obesity [34,58]	State	Most risk factors have an estimate based on a large scale household survey‡ 'Lack of physical activity' measured by household survey conducted in 6 states||
			
		Tobacco & alcohol use [34,42,53,58]	State	
			
		Fruit and vegetable intake [32,34,58,59]	State	
			
		Lack of physical activity [34]	State||	
			
		Self-reported diagnosis of diabetes [34,58]	State	
	
	**Service provision**	Self-reported treatment of disease [34]	State||	Estimate based on household survey conducted in 6 states||

**4. Diarrhoeal diseases **(5.1%)	**Mortality**	Percent of adult deaths attributed to diarrhoea [34]	National	Estimate based on a small sample of verbal autopsy on sibling deaths§
	
	**Morbidity & health status**	Prevalence of diarrhoea in children [34,38,58-61,63]	State	Most estimates based on data from large scale household surveys‡
			
		Self-reported hospitalisation for diarrhoea [33]	National	
	
	**Risk factors**	Access to clean water and sanitation [19,33-35,37-41,58-60,63,64]	District	Most estimates based on data from large scale household surveys‡
			
		Prevalence of childhood underweight [58-60,62]	State	
			
		Vitamin A deficiency in children [38]	State	
			
		Percentage of children receiving food rich in vitamin A in last 24 hrs [58]	State	
			
		Breastfeeding practices [38,58,63]	District	
	
	**Service provision**	Treatment of childhood diarrhoea [38,58-61,63]	State	Most estimates based on large scale household surveys‡
			
		Child vitamin A supplementation [38,43,58,59,61,63]	District	

**5. Unipolar depression** (4.9%)	**Morbidity & health status**	Self-reported diagnosis of depression [34]	State||	Estimate based on household survey conducted in 6 states||
	
	**Risk factors**	Alcohol use [34,53,58]	State	Both risk factors estimated by at least one large scale household survey‡
			
		Prevalence of women who experienced childhood sexual abuse [58]	National	
	
	**Service provision**	Self-reported treatment coverage [34]	State||	Estimate based on household survey conducted in 6 states||

**6. HIV/AIDS** (3.4%)¶	**Mortality**	None*		
	
	**Morbidity & health status**	HIV prevalence [8,58]	State	Estimate based on sentinel surveillance and large scale household survey‡
	
	**Risk factors**	Unsafe sex [51,58]	State	Both risk factors estimated by large scale household survey‡ Unsafe sex also estimated by behavioural surveillance
			
		Use of clean injections [58]	State	
	
	**Service provision**	Coverage of treatment with antiretrovirals [48,65]	State	Estimates mostly based on large scale household surveys and behavioural surveillance‡
			
		HIV testing uptake [34,51,58]	State	
			
		Condom use [34,51,58]	State	

**7. Maternal conditions** (2.9%)	**Mortality**	Maternal mortality ratio [59,60,66]	State	Maternal mortality ratio and cause of death estimates based on sample registration system data A large scale household survey has also estimated maternal mortality ratio‡
			
		Cause of maternal death [66]	National	
			
		Percent of total female deaths attributed to maternal conditions [34]	National	
	
	**Morbidity & health status**	Prevalence of postpartum complications [58]	State	Both estimates based on large scale household survey‡
			
		Prevalence of delivery complications [63]	State	
	
	**Risk factors**	Anaemia among women [58,59,62]	District	Most estimates based on data from large scale household survey‡
			
		Underweight among women [34,58,59]	State	
			
		Contraception non-use [38,43,58-61,63]	District	
			
		Birth spacing [58-60]	State	
			
		Number of children ever born 5 or more [58-61,63]	District	
			
		Vitamin A deficiency in pregnant women [58,61,63]	National	
			
		Antenatal care received [33,34,38,41,43,58-61,63]	District	
			
		Skilled birth attendance [33,34,38,41,58-61,63]	District	
			
		Low birth weight [38,58,59,61]	District	
			
		Prevalence of postpartum complications [58-61,63]	District	

	**Service provision**	Contraception coverage [38,43,58-61,63]	District	Most estimates based on data from large scale household survey‡
			
		Antenatal care coverage [33,34,38,41,43,58-61,63]	District	
			
		Women receiving iron supplementation [38,41,43,58-61,63]	District	
			
		Skilled birth attendance coverage [33,34,38,41,58-61,63]	District	
			
		Postnatal care coverage [33,38,41,58,59]	State	

**8. Tuberculosis **(2.8%)	**Mortality**	Percent of adult deaths attributed to tuberculosis [34]	National	Estimate based on a small sample of verbal autopsy on sibling deaths§
	
	**Morbidity & health status**	Self-reported tuberculosis diagnosis [34,58-60,63]	State	Most estimates based on large scale household surveys‡
			
		Self-reported hospitalisations for tuberculosis [33]	National	
	
	**Risk factors**	Tobacco & alcohol use [34,42,53,58]	State	Most estimates based on large scale household surveys‡
			
		Type of cooking fuel used [19,34,38,40,58,59,63]	District	
			
		Malnutrition [32,34,38,58,60,62]	District	
	
	**Service provision**	Annualised case detection rate for new smear positive cases [45]	District	Most estimates based on tuberculosis surveillance system Self-reported tuberculosis treatment estimates mostly based on large scale household surveys‡
			
		Proportion of new sputum positive out of total new pulmonary cases [45]	District	
			
		Smear conversion rate [45]	District	
			
		Treatment success rate [45]	District	
			
		Self-reported tuberculosis treatment [34,58,59,63]	State	

**9. Cerebrovascular disease** (2.5%)	**Mortality**	Percent of adult deaths attributed to paralysis [34]	National	Estimate based on a small sample of verbal autopsy on sibling deaths§
	
	**Morbidity & health status**	None*		
	
	**Risk factors**	Prevalence of overweight & obesity [34,58]	State	Most risk factors have an estimate based on a large scale household survey‡ 'Lack of physical activity' measured only by household survey conducted in 6 states|
		
		Tobacco & alcohol use [34,42,53,58]	State	
		
		Fruit and vegetable intake [32,34,58,59]	State	
		
		Lack of physical activity [34]	State||	
		
		Self-reported diagnosis of diabetes [34,58]	State	
	
	**Service provision**	*None*		

**10. Cataract** (2.2%)	**Morbidity & health status**	Prevalence of blindness [36,60,63]	State	Estimates based on both large scale household survey and household survey conducted in 6 states ‡ ||
			
		Previous diagnosis of cataracts [34]	State||	
			
		Self-reported hospitalisations for cataracts [33]	National	
	
	**Risk factors**	Tobacco smoking [34,42,53,58]	State	Estimates based on of large household surveys, household survey conducted in 6 states and sample registration system ‡ ||
			
		Self-reported diagnosis of diabetes [34,58]	State	
	
	**Service provision**	Number of cataract operations performed [44]	State	Estimates based on household survey conducted in six states and service generated data||
			
		Those with previous diagnosis who have accessed surgery [34]	State||	

**11. Road traffic injuries** (2.1%)	**Mortality**	Deaths from road traffic injuries [25]	State	Estimate based on surveillance
	
	**Morbidity & health status**	Prevalence of road traffic injury in last one year [34]	National	Estimate based on household survey conducted in 6 states ||
	
	**Risk factors**	Alcohol use [34,53,58]	State	Estimates based on both large scale household survey and household survey conducted in 6 states‡ ||
	
	**Service provision**	Those with previous road traffic injury who had accessed emergency care [34]	State||	Estimate based on household survey conducted in 6 states ||

*Other than that generated by modelling and projections

†Only the latest National Family Health Survey provides the perinatal mortality rate specifically, the earlier surveys and the sample registration system give the infant mortality rate.

‡The large scale household surveys include: National Family Health Survey (2005/6 survey sampled 109,041 households), Reproductive and Child Health District Level Household Survey (2002/4 survey sampled 620,107 households), National Sample Survey on morbidity, health care and the condition of the aged (sampled 73,868 households), National Sample Survey on nutritional intake in India (sampled 124,644 households) and United Nations Children's Fund Multiple Indicator Survey (sampled 119,305 households).

§The World Health Survey examined a total of 1954 deaths of which cause of death was attributed to 55% of female and 61% of male deaths.

||The World Health Survey was conducted in six states and state level estimates available only for the states of Assam, Karnataka, Maharashtra, Rajasthan, Uttar Pradesh, West Bengal; national pooled estimates also available; sampled 10,729 households.

¶ The estimated burden of HIV/AIDS in India was reduced recently to about half of the previous estimate based on new population-based data [67]; the estimated DALYs lost in 2002 shown in this table is therefore an overestimate.

Mortality information generated by the essential sources other than modelled projections is available to some degree for the leading causes of disease burden in India except lower respiratory tract infections and HIV/AIDS. However, it is not complete for many conditions. For example, the perinatal mortality rate is available but there are no data available on the causes of perinatal death. Additionally, for many of the conditions, the only mortality information available has been generated by the World Health Organisation World Health Survey, which used verbal autopsy for sibling deaths. These estimates have limitations in the detail of the cause of death, the small sample of deaths examined, and the fact that for 46% of female deaths and 40% of male deaths the cause of death was not determined [34]. This lack of primary data on mortality is being partly addressed by the addition of verbal autopsy to the sample registration system, which is likely to provide mortality data for all major causes in India [6].

There are more sources generating information on morbidity for maternal and child health and communicable diseases as compared with non-communicable diseases. All of the available morbidity information is sourced from self-reporting of conditions in household surveys. The only exception to this is HIV prevalence. Although, self-reporting of conditions in surveys is problematic, use of biological tests in surveys for the estimation of population prevalence can be tedious for many conditions [4]. The use of other sources such as service records can aid to fill some of these gaps, although these estimates are prone to bias [4]. As with mortality information, modelled morbidity estimates for India have been produced by the Global Burden of Disease and Risk Factors study [15]. Additionally, the National Commission on Macroeconomics and Health produced modelled morbidity estimates for tuberculosis, HIV/AIDS, diarrhoeal diseases, blindness, mental health and cardiovascular disease [57].

Risk factor information is generated by a large number of essential sources in addition to the estimates by the Global Burden of Disease and Risk Factors Study. The majority of this information is generated by household surveys. Information on risk factors is more substantial for perinatal and maternal conditions than for the other conditions. The proposed Integrated Disease Surveillance Project is expected to enhance the risk factor information for non-communicable diseases and injuries, but outputs from this initiative are not available yet [9].

Service provision information is lacking for the leading non-communicable diseases and road traffic injuries. Only one essential source generates information on services for ischaemic heart disease, unipolar depression and road traffic injuries, and these estimates are based on data from a household survey, held in only 6 states in India [34]. There is no information on services for cerebrovascular disease.

Overall, there is a significant lack of relevant information on non-communicable diseases and injuries, which now account for a major proportion of the disease burden in India [15]. Household surveys are the main source of primary information on the leading causes of disease burden in India. Their main focus is on communicable diseases and maternal and child health. The other essential sources from which information is available on the leading causes of disease burden in India are a surveillance system [8], service generated data [43-45], sample registration system [6], behavioural surveillance [51,53] and census [19].

### Geographical level of information

The majority of information produced by the essential sources is reported at the state level (Tables 2). Census produces information at the town and city level including demographic information, access to clean water, sanitation and use of cooking fuels [19]. The National Cancer Registry Programme generates information mainly at the city level [26]. The Reproductive and Child Health District Level Household Survey was designed to provide monitoring of the Reproductive and Child Health programme at the district level[29]. The Bulletin on Rural Health Statistics generates some information at the district level on numbers of public health facilities in each district [46]. The Revised National Tuberculosis Control Programme provides performance indicators for the programme at the district level [45].

### Availability in the public domain

The availability of the information produced by the essential sources in the public domain on the internet was variable (Table 2). The sources that had all information freely available included the National Polio Surveillance Project [27], Reproductive and Child Health District Level Household Surveys [29], National Family Health Surveys [30], World Health Survey [34], Revised National Tuberculosis Control Programme [45], Reproductive and Child Health Facility Survey [49], national health accounts [54] and the Global Burden of Disease and Risk Factor Study [16]. In addition, National Health Profile [47], Jansankhya Sthirata Kosh health maps [50], Ministry of Health and Family Welfare "Annual Report" [55], Planning Commission "Five Year Plan" [56] and the National Commission on Macroeconomics and Health [57] had reports freely available. The sources which required the purchase of microdata include all National Sample Surveys [31-33,35-37,39,41,42]. Only some information was available for a variety of essential sources, more specifically the census [19], monitoring of births and deaths [6,20], four surveillance and response systems [8,25,26,28], two household surveys [38,40], two source of service generated data [43,44], two under mapping of health facilities [46,48] and three behavioural surveillance systems [51-53]. The main reason for these sources not scoring well was the non-availability of reports or microdata. For example, the UNICEF Multiple Indicator Survey has a report available, but no microdata. The Integrated Disease Surveillance Project had the lowest score for data available in the public domain.

## Discussion

Ready availability of essential health information is imperative for the development of informed and effective systems for improving health of societies. This paper provides a broad overview of the data readily available in the public domain on the internet related to essential health information in India. It highlights a number of issues that need to be addressed to improve the scope and availability of health information in India.

There is a lack of primary data on mortality and cause of death information for the majority of the leading causes of disease burden in India. While there are modelled estimates of causes of death generated by the Global Burden of Disease and Risk Factors project, there are minimal primary data on causes of death. These data would normally be generated by a complete death registration system, which is not present in India. The national birth and death registration system is estimated to cover about half of deaths in India [20]. The recent addition of verbal autopsy to the sample registration system is expected to provide all-cause mortality information to some degree in the near future [6]. The Integrated Disease Surveillance Project is also expected to contribute to this information, although no data are available yet [9].

There were substantial gaps in the available information on non-communicable diseases and injuries. This is significant as the epidemiological transition is well underway in India. Whereas previously maternal and child conditions and communicable diseases where responsible for the majority of the disease burden, more recently the rising burden of non-communicable diseases is being documented in India and is projected to increase [68,69]. While there is still need for information on maternal and child conditions and communicable diseases, there is also now additional need for information on non-communicable diseases and injuries. There have been some recent efforts to address this gap. The Integrated Disease Surveillance Project is planned to include surveillance of risk factors for non-communicable diseases and information on road traffic accidents [9]. The sample registration system is also expected to contribute information on a number of non-communicable disease risk factors [6].

Information on the health infrastructure and human resources is not complete as it focuses primarily on the public health system, while in the most recent National Family Health Survey 65% of the households in India reported seeking health care from the private sector [58]. Information is available for the public health system at all levels of health care including primary care and detailed distribution of human resources is also available. However, information available on the private health sector is restricted to the number of hospital beds and estimates of the number of entire health workforce. These estimates of the total number of health workers are inadequate [70] and do not allow a good understanding of the distribution of the health workers within the private sector.

The lowest level for which the majority of the information generated by the essential sources is available is the state level. Although this information is useful, there is are wide variations between districts within the states. For example, the districts of Agra and Rampur in the state of Uttar Pradesh (population 184 million [19]) have literacy rates of 63% and 39% respectively [19]. In the state of Andhra Pradesh, 69% of the villages in West Godavari district have a medical facility whereas only 46% of the villages have a medical facility in Chittoor district [19]. Therefore, information at the lower administrative levels is especially important in a large country like India. Furthermore, with the emphasis on decentralisation of health services in India [57], district level information is needed. The introduction of the Reproductive and Child Health District Level Household Surveys recognises this need, however these surveys only monitor maternal and child health [29]. It should be noted that there may be information available at the district level to administration and management officials, which has not been captured in this review as it is not readily available in the public domain.

The availability in the public domain of the information generated by the essential sources varied. The ready availability of health information, including primary data, informs a range of actions to improve population health and the health system and thus there is an increasing momentum for it to be available in the public domain [3]. There are some considerations which need to be taken into account when making data available in the public domain, one of which is maintaining confidentiality. The availability of data sets in the public domain from over 150 demographic and health surveys, including those for India, demonstrates that this is feasible [3].

A limitation of our analysis is that it covers information on the essential sources readily available in the public domain on the internet. However, as stated above, the ready availability of information generated by the health information system is crucial for use by all stakeholders to efficiently improve population health. Therefore, the findings in this paper are significant in highlighting what health information is readily available in the public domain in India and what is not, which would help bring attention to the major deficiencies that exist currently. Although we did extensive web searches for the essential sources of health information that were available in the public domain, it is possible that we could have missed some sources that were not readily available. However, it seems unlikely that this would have led to a substantially different message from this paper.

One of the essential sources, health research, was not examined in this paper [10]. The contribution of research to health information cannot be overstated as it often fills gaps in the information generated by the other sources and can guide conceptual development of health policy and systems [71].

All essential sources of health information complement each other to together produce a full picture of the health of a population. Thus a coordinated overall approach should be taken when strengthening health information systems, taking into account all essential sources. This will aid the effective use of scarce resources [4]. It has been suggested that streamlining of surveys and careful planning of a national survey programme will ensure all priority health topics are covered and costs minimised by avoiding duplication [4]. For example, there is overlapping information produced by a variety of sources on reproductive and child health in India. If this were streamlined the resources saved could be utilised to generate the basic health information that is missing for non-communicable diseases and injuries.

The essential sources and the information they produce are just one component of a national health information system. The policy and leadership environment, infrastructure, the information dissemination and utilisation are the other important aspects of a national health information system [72]. A comprehensive framework for the assessment of all these components has recently been developed by the WHO Health Metrics Network [73]. The findings reported in this paper provide an initial broad understanding of the essential health information readily available in the public domain in India. Further detailed understanding of the major gaps identified would be needed in order to develop strategies to address them for strengthening the health information system of India.

## Conclusion

This broad overview of the essential health information readily available in the public domain on the internet for India identified several weaknesses such as the lack of information on non-communicable diseases and injuries, primary data on causes of death, the private health sector and district level information. While some recent initiatives will help enhance the health information system of India, a systematic approach is needed to develop a streamlined system that addresses the critical gaps. Further nuanced assessment of the gaps in the health information system of India is needed in order to inform its further development.

## Competing interests

The authors declare that they have no competing interests.

## Authors' contributions

MZR, RD and LD contributed to the design of this research, analysis and interpretation, and approved the final version of the paper. This research contributed to the Master of International Public Health Honours thesis by MZR at the University of Sydney which was supervised by RD and LD. All authors read and approved the final manuscript.

## Pre-publication history

The pre-publication history for this paper can be accessed here:


